# Phylogenomic Analyses Reveal Species Relationships and Phylogenetic Incongruence with New Member Detected in *Allium* Subgenus *Cyathophora*

**DOI:** 10.3390/plants14132083

**Published:** 2025-07-07

**Authors:** Kun Chen, Zi-Jun Tang, Yuan Wang, Jin-Bo Tan, Song-Dong Zhou, Xing-Jin He, Deng-Feng Xie

**Affiliations:** Key Laboratory of Bio-Resources and Eco-Environment of Ministry of Education, College of Life Sciences, Sichuan University, Chengdu 610065, China; captainfxh@126.com (K.C.); 18723052386@163.com (Z.-J.T.); wang_yuan2000@163.com (Y.W.); jinbotan@scu.edu.cn (J.-B.T.); zsd@scu.edu.cn (S.-D.Z.); xjhe@scu.edu.cn (X.-J.H.)

**Keywords:** subgenus *Cyathophora*, phylogenome, phylogeny, phylogenetic discordance, reticulate evolution

## Abstract

Species characterized by undetermined clade affiliations, limited research coverage, and deficient systematic investigation serve as enigmatic entities in plant and animal taxonomy, yet hold critical significance for exploring phylogenetic relationships and evolutionary trajectories. Subgenus *Cyathophora* (*Allium*, Amayllidaceae), a small taxon comprising approximately five species distributed in the Qinghai–Tibet Plateau (QTP) and adjacent regions might contain an enigmatic species that has long remained unexplored. In this study, we collected data on species from subgenus *Cyathophora* and its close relatives in subgenus *Rhizirideum*, as well as the enigmatic species *Allium siphonanthum*. Combining phylogenomic datasets and morphological evidence, we investigated species relationships and the underlying mechanism of phylogenetic discordance. A total of 1662 single-copy genes (SCGs) and 150 plastid loci were filtered and used for phylogenetic analyses based on concatenated and coalescent-based methods. Furthermore, to systematically evaluate phylogenetic discordance and decipher its underlying drivers, we implemented integrative analyses using multiple approaches, such as coalescent simulation, Quartet Sampling (QS), and MSCquartets. Our phylogenetic analyses robustly resolve *A. siphonanthum* as a member of subg. *Cyathophora*, forming a sister clade with *A. spicatum.* This relationship was further corroborated by their shared morphological characteristics. Despite the robust phylogenies inferred, extensive phylogenetic conflicts were detected not only among gene trees but also between SCGs and plastid-derived species trees. These significant phylogenetic incongruences in subg. *Cyathophora* predominantly stem from incomplete lineage sorting (ILS) and reticulate evolutionary processes, with historical hybridization events likely correlated with the past orogenic dynamics and paleoclimatic oscillations in the QTP and adjacent regions. Our findings not only provide new insights into the phylogeny of subg. *Cyathophora* but also significantly enhance our understanding of the evolution of species in this subgenus.

## 1. Introduction

*Allium* L., a dominant monocotyledonous genus, represents the sole member in the tribe Allieae (Amaryllidaceae) [1]. This genus comprises over 1000 recognized species predominantly distributed across the Northern Hemisphere [2,3] exhibiting remarkable habitat plasticity, ranging from xeric well-drained soils to hydric environments, and even growing in swampy or aquatic conditions [4]. This extensive geographical distribution coupled with habitat heterogeneity has fostered both high species richness and remarkable morphological diversification within this genus [5,6,7]. Many species are characterized by bulbs enclosed in membranous (sometimes fibrous) tunics, free or almost-free tepals, and often a sub-gynobasic style, and are frequently accompanied by strong or distinctive smells and tastes [6]. Previous studies have suggested that this genus includes two centers of species diversity: one spanning Southwest/Central Asia to the Mediterranean, and another in North America [5,6,8,9]. Although extensive studies have established a robust phylogenetic framework for *Allium* species, which has classified this genus into 15 subgenera [6], and recent advances in sequencing technologies have further facilitated the investigations of species relationships and evolution within subgenera or sections [10,11,12,13,14,15,16], significant knowledge gaps persist in several subgenera. These include unresolved species composition, ambiguous interspecific relationships, and underexplored evolutionary process within specific taxonomic groups. Particularly, for taxonomically neglected species that have long been understudied, their phylogenetic placement and evolutionary relationships with closely related taxa necessitate comprehensive investigation and systematic resolution.

Subgenus *Cyathophora* R.M. Fritsch is a small taxon of genus *Allium* L., currently comprising five species: *Allium mairei* H.Lév., *A. spicatum* (Prain) N. Friesen, *A. farreri* Stearn, *A. cyathophorum* Bureau & Franch, and *A. tetraploideum* M.J. Li & X.J. He [16,17]. All species of this subgenus are distributed across the Qinghai–Tibet Plateau (QTP) and adjacent regions. Notably, *A. spicatum* [18], distinguished by its unique spicate inflorescence, is restricted to the arid western QTP, whereas the remaining four species with umbellate inflorescences primarily occur in the Hengduan Mountains (HDMs) and surrounding regions [10]. Since the establishment of this subgenus, numerous studies have been conducted. Molecular evidence indicates that *A. spicatum* belongs to subg. *Cyathophora*, despite its conspicuously elongated spike which contrasts with the typically umbel or capitate inflorescences found in most *Allium* species [6,18]. Li et al., 2019 [17], reclassified *A. cyathophorum* var. *farreri* as a distinct species, *A. farreri*, based on integrated evidence from morphology, cytology, and molecular biology. Phylogeographic studies indicate that the origin of this subgenus coincided with the uplift of the Hengduan Mountains (HMDs) around 4–3 Ma, with subsequent Quaternary climatic fluctuations intensifying species divergence and shaping current distribution patterns [10,19]. Subsequent phylogenetic analyses demonstrate the following: (i) *A. mairei* represents the earliest diverged lineage within this subgenus; (ii) *A. spicatum* forms a sister clade to *A. farreri*; (iii) *A. cyathophorum* shows sister relationships with the tetraploid *A. tetraploideum*, which originated from hybridization between at least two extant diploids (*A. farreri* and *A. cyathophorum*) and an extinct diploid progenitor [11,16].

However, species diversity within subg. *Cyathophora* may exceed that reported for the five species in previous studies, and the phylogenetic relationships within this subgenus might differ from existing interpretations. These reassessments stem from our investigation of *Allium siphonanthum* J.M Xu in this study. Originally described in *Flora Reipublicae Popularis Sinicae* [20], *A. siphonanthum* has received minimal scientific attention since its discovery, with only *the Flora of China* documenting its narrow distribution in Zhongdian, Yunnan Province, China. To date, only four specimens of this species have been collected. Although Li Q.Q., 2010 [21], classified it within subg. *Rhizirideum*, this assignment was made without any morphological or molecular data specific to *A. siphonanthum*. Here, our phylogenomic analyses conclusively assign *A. siphonanthum* within subg. *Cyathophora*. Consequently, the phylogenetic placement of *A. siphonanthum*, interspecific relationships within this subgenus, and associated evolutionary questions require comprehensive re-evaluation.

Recent advances in sequencing technologies and bioinformatics have facilitated the widespread adoption of phylogenomic methods in species taxonomy and evolutionary biology, leveraging phylogenomic data’s rich informative characters to enhance phylogenetic resolution across diverse lineages [22,23,24,25,26,27,28]. These data and associated analytical approaches have demonstrated their usefulness not only in resolving taxonomic classifications but also in reconstructing phylogenetic relationships and elucidating evolutionary patterns within the genus *Allium* [11,13,14,16,25,29,30]. Here, we collected all currently recognized species within subg. *Cyathophora* and phylogenetically proximate species from subg. *Rhizirideum*, including the taxonomically contentious *A. siphonanthum*. Through the integration of morphological characteristics, transcriptomic profiles, and whole-genome resequencing datasets, we constructed a phylogenomic framework to achieve the following: (i) reconstruct high-resolution phylogenies for subg. *Cyathophora*, thereby determining *A. siphonanthum*’s systematic position and delineating its phylogenetic affinities; (ii) elucidate the evolutionary mechanisms driving the observed phylogenetic discordance within this subgenus.

## 2. Results

### 2.1. Summary of Morphological Characteristics

By comparing morphological characteristics among species within subg. *Cyathophora*, we identified significant differences (Table 1). *Allium cyathophorum*, *A. tetraploideum*, and *A. farreri* exhibit remarkably similar morphology, differing only in scape shape, perianth color, and ploidy level: *A. tetraploideum* has three-angled scapes (vs. two-angled in others), dark maroon perianths (vs. purple in *A. cyathophorum* and *A. farreri*), and is tetraploid (vs. diploid). *A. farreri* is distinguished by acuminate perianth segments and triangular basal appendages on the inner filaments. *Allium siphonanthum* displays diagnostic traits: subreticulate bulb tunics, globose, densely multiflowered umbel, two-valved spathes, inner filaments broadened baselly with entire or one-toothed margins, and styles exceeding the ovary. *A. spicatum* is unique within the subgenus due to its spike inflorescence, characterized by pedicels much shorter than perianths and dense flowers. It also possesses one-valved spathes, white to purple-red perianths (similar to *A. siphonanthum*), and styles longer than the ovary, but differs in bearing inner filaments that are either entire or two-toothed basally on each side. *Allium mairei* is diagnosed by its umbel inflorescence bearing very few flowers. Additionally, it usually has clustered bulbs, two-angled scapes, pedicels of unequal length that are 1.5–2 times longer than the perianths, a one-valved spathe, and styles that are shorter than the ovary.

### 2.2. Transcriptome Assembly and Phylogeny Results

De novo transcriptome assemblies were generated for all *Cyathophora* species and relatives in subgenus *Rhizirideum*, yielding assemblies with total base counts ranging from 19,713,114 to 28,906,896 bases with mean transcript lengths spanning 782–892 bp. Transcript quantification revealed interspecific variation in assembly complexity, exhibiting total counts from 28,042 (*A. spicatum*) to 35,307 (*A. siphonanthum*), while transcript N50 values, indicating scaffold continuity, ranged from 997 bp (*Allium mairei* H.Lév.) to 1245 bp (*Allium mongolicum* Regel) (Table S1).

**Figure 1:**
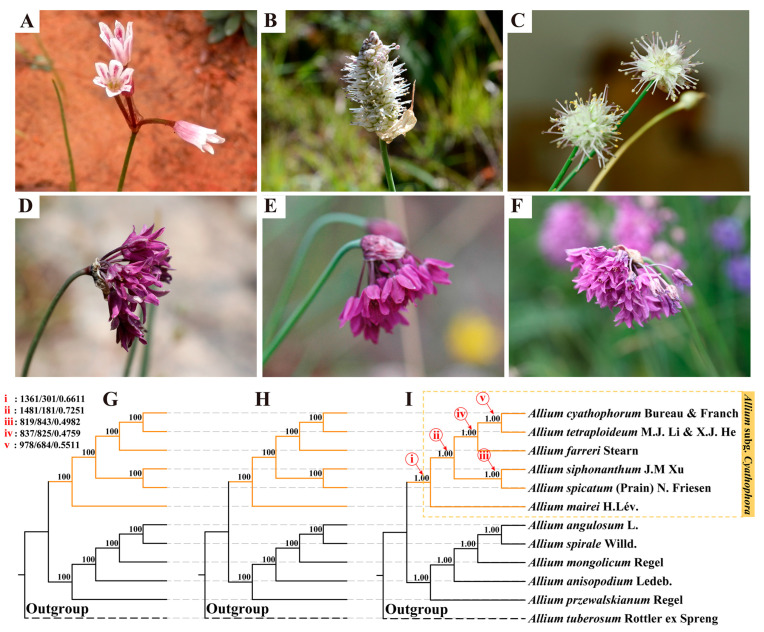
The inflorescences characteristics (**A**–**F**) and the phylogenetic relationships (**G**–**I**) of species in subgenus *Cyathophora*. (**A**) *A. mairei*; (**B**) *A. spicatum*; (**C**) *A. siphonanthum*; (**D**) *A. farreri*; (**E**) *A. tetraploideum*; (**F**) *A. cyathophorum*. The concatenation- and coalescent-based species trees of subg. *Cyathophora* species based on 1662 SCGs were inferred from (**G**) IQ-TREE and (**H**,**I**) ASTRAL-III. The support values from the different methods [i.e., UFBS/IQ-TREE, BS/ASTRAL-III, and LPP/ASTRAL-III] are indicated above their respective branches. The yellow dotted box represents the subg. *Cyathophora*. The critical evolutionary nodes with red colors (i–v) display the number of gene trees in concordance/conflict with the display nodes and the ‘Internode Certainty All’ (ICA) scores.

Phylogenetic reconstruction using concatenation and coalescent approaches based on 1662 single-copy genes (SCGs) yielded congruent topologies (Figure 1), consistently resolving *A. mairei* as the earliest diverging lineage within subg. *Cyathophora*. The analyses strongly supported (UFBS/BS = 100; LPP/BS = 1.00/100) a sister relationship between *A. spicatum* and *A. siphonanthum*, with *A. cyathophorum* forming a clade sister to *A. tetraploideum*, which subsequently showed derived affinity to *A. farreri*. All subg. *Cyathophora* species formed a monophyletic clade that was reciprocally sister to subg. *Rhizirideum*. Internode certainty analysis (ICA) revealed discordance between the species tree and individual gene trees (Figure 1 and Figure S1). More than 50% of the 1662 gene trees supported core divergence events within *Cyathophora*, except for the diverged node of *A. spicatum* and *A. siphonanthum* (Figure 1, node (iii), 819 supporting trees, ICA = 0.498). Node-specific concordance varied substantially across the phylogeny, with the numbers of concordant gene trees ranging from 837 (node (iv)) to 1361 (node (i)), and ICA values ranging from 0.4759 to 0.7251.

### 2.3. Phylogenetic Results Based on Plastome Data

**Figure 2:**
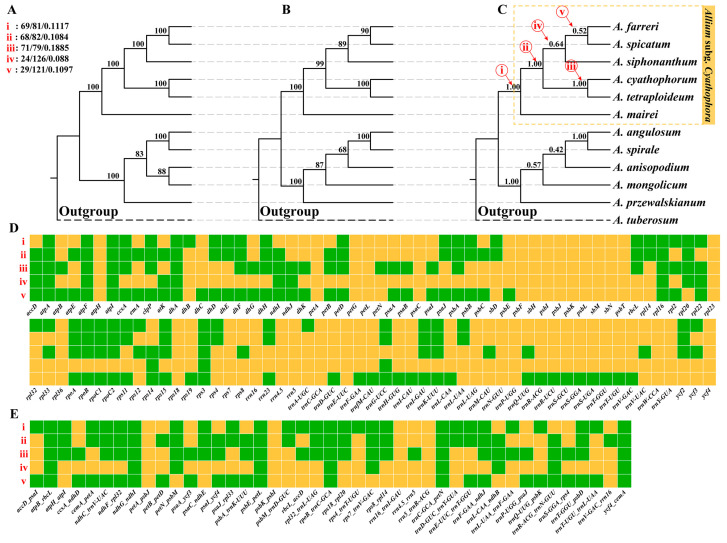
Phylogenetic trees based on 150 plastid loci. (**A**–**C**) The species tree was inferred from IQ-TREE and ASTRAL-III, and the numbers on internal branches represent the UFBS/IQ-TREE, BS/ASTRAL-III, and LPP/ASTRAL-III, respectively. The yellow dotted box represents the subg. Cyathophora. The critical evolutionary nodes (marked with i–v in red) display the number of gene trees in concordance/conflict with the display nodes and the ‘Internode Certainty All’ (ICA) scores. (**D**) A heatmap showing the comparative results for each of the 107 gene trees with the species trees at the five nodes (i–v), with the gene trees exhibiting concordant and conflict topologies marked with green and orange squares, respectively. (**E**) A heatmap showing the comparative results for each of the 43 IGS trees to the species trees at the five nodes (i–v), with the IGS trees exhibiting concordant and conflict topologies marked with green and orange squares, respectively.

The plastid genomes (plastomes) of subg. *Cyathophora* species exhibited a size range of 152,876 bp to 153,956 bp (Table S2). A total of 107 genes and 43 intergenic spacer regions (IGSs) were selected for phylogenetic reconstruction. The concatenated alignment of 150 loci spanned 112,586 bp, containing 3935 variable sites with 1149 parsimony-informative characters. Both the concatenation- and coalescent-based approaches produced congruent phylogenetic topologies (Figure 2 and Figure S2). However, the plastid-derived phylogeny revealed contrasting relationships with transcriptome-based inferences: (i) *Allium mairei* occupied the basal divergent position; (ii) *Allium cyathophorum* and *A. tetraploideum* formed a sister clade; (iii) *A. farreri* was sister to *A. spicatum*, and this clade was sister to *A. siphonanthum*. All the nodal relationships received strong statistical support (UFBS/BS = 100; LPP/BS > 0.9/90), except for the *A. farreri* + *A. spicatum* + *A. siphonanthum* clade, where the local posterior probabilities were moderate (LPP < 0.7).

Phylogenetic conflict analysis using ICA scores revealed statistically significant incongruence among plastid gene trees at key nodes within subg. *Cyathophora*. Of the 150 plastid loci analyzed, 69 loci supported the divergence node of *A. mairei* (Figure 2, node (i), ICA = 0.1117), 68 loci supported the origin of the *A. cyathophorum + A. tetraploideum* clade (Figure 2, node (ii), ICA = 0.1084), and 71 loci supported the divergence between *A. tetraploideum* and *A. cyathophorum* (Figure 2, node (iii), ICA = 0.1885). In contrast, the divergence nodes of the remaining species showed markedly lower support, with fewer than 30 loci supporting them and their associated ICA values ranging from 0.0880 to 0.1097 (Figure 2, nodes (iv), (v)).

### 2.4. Concordance Analysis

**Figure 3:**
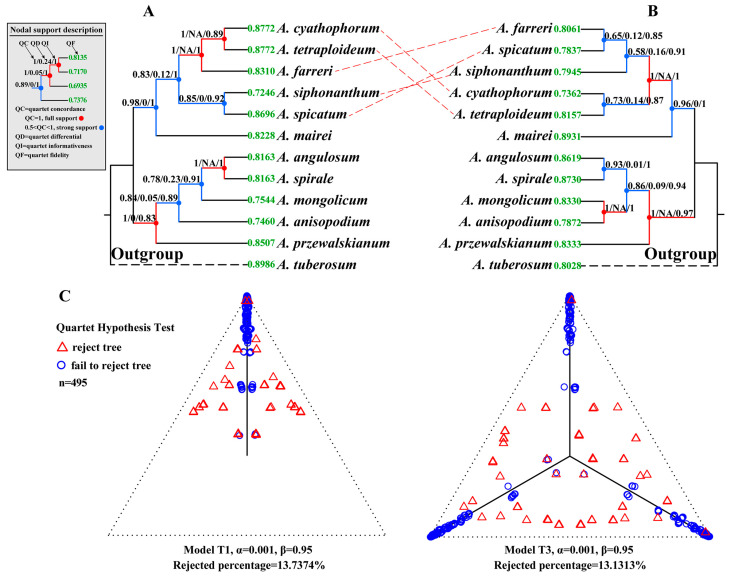
Phylogenetic discordance (**A**,**B**) and ILS detection (**C**). The species trees are inferred from (**A**) 1662 SCGs and (**B**) 150 plastid loci. The nodal support values are explained by referring to the ‘Nodal support description’ for a visual guide; the nodes show QC/QD/QI values in black above their branches, the terminals display QF values in green at the branch tips. The red branches represent full support with QC = 1; blue represents strong support, with QC values from 0.5 to 1. (**C**) Simplex plots of qcCFs under the MSC model of ILS with the T1 and T3 models (α = 0.001, other levels are provided in Figure S5) for the 1662 SCG trees. The red triangles denote MSC model rejection, while the blue circles indicate ILS-driven phylogenetic discordance.

**Figure 4:**
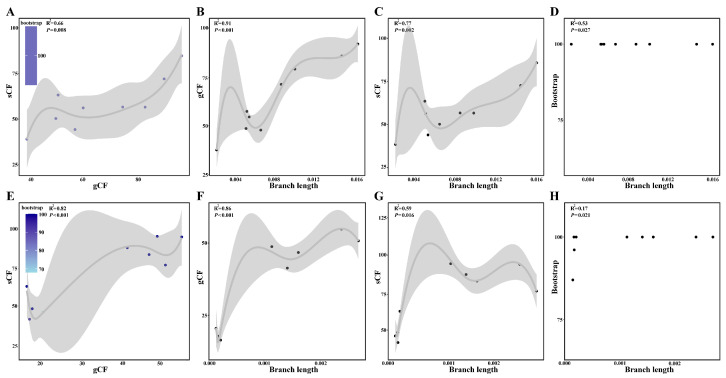
Correlative relationships between concordance factors (gCF and sCF) and bootstrap (BS) values and branch lengths at all phylogenomic nodes, examined through analyses of (**A**–**D**) SCG dataset and (**E**–**H**) plastid loci dataset.

Through the comparative analysis of the species trees reconstructed from the SCGs and plastid genomes, we detected substantial phylogenetic incongruences within subg. *Cyathophora* (Figure 3). The SCG-derived species tree resolved *A. siphonanthum* as sister to *A. spicatum*, with *A. farreri* forming a clade with *A. cyathophorum* and *A. tetraploideum* (Figure 3A). In contrast, the plastid phylogeny showed different relationships: *A. siphonanthum* clustered with *A. farreri* + *A. spicatum*, while *A. cyathophorum* appeared as sister to *A. tetraploideum* (Figure 3B). To further investigate the causes of gene tree discordance and cytonuclear incongruence, we conducted two additional analyses. First, we calculated two concordance metrics: the gene concordance factor (gCF) and the site concordance factor (sCF). In nuclear datasets, gCF values (ranging from 38.5% to 95.4%; mean = 65.89%) consistently exceeded sCF values (38.0–88.9%; mean = 59.24%) (Figure S3). In contrast, the plastid tree showed lower gCF values (16.7–64.7%; mean = 34.96%) but higher sCF values (42.4–95.1%; mean = 71.08%) (Figure S4). Significant correlations (*p* < 0.05) were observed between concordance factors (gCF and sCF) and branch length/bootstrap support (BS) across both single-copy genes (SCGs) and plastid datasets (Figure 4). Reduced gCF and sCF values consistently corresponded to shorter branches or lower BS. Notably, most nodes in both SCGs and plastid trees exhibited maximum BS (100%), with branch length positively correlating with BS (Figure 4D,H).

Quartet Sampling (QS) analysis further revealed universally high quartet concordance (QC) across all nodes, with QC values exceeding 0.50. The mean QC values reached 0.92 (SCGs-based tree) and 0.85 (plastid tree), respectively. Notably, four nodes in the SCGs-based tree and three nodes in the plastid tree demonstrated maximum QC support (QC = 1.0) (Figure 3A,B). Most nodes exhibited a characteristic pattern of high QC (1.0), low/absent quartet discordance (QD: 0-NA), and complete quartet informativeness (QI: 1.0). This signature pattern (QC/QD/QI = 1/0/1 or 1/NA/1) indicates strong support for the inferred topology at these nodes based on Quartet Sampling. Both trees showed consistently high quartet fidelity (QF >0.70), ranging from 0.7246 to 0.8986 in the SCGs-based tree and 0.7362 to 0.8931 in plastid tree. These findings collectively indicate strong topological consistency across the quartets, with no evidence of problematic taxa.

### 2.5. Detection of ILS

Phylogenetic analyses using MSCquartets evaluated incomplete lineage sorting (ILS) intensity within subg. *Cyathophora* by applying the T1 and T3 models to the 1662 single-copy gene (SCG) trees. The analysis identified 495 exhaustive quartets across the taxa. Under increasing rejection thresholds (0.01 to 1 × 10^−6^), the proportion of rejected trees decreased from 18.58% to 9.29% (T1 model) and 17.57% to 8.28% (T3 model) (Figure 3C and Figure S5). Simplex plots revealed the dense clustering of qcCF markers along the T1/T3 model trajectories, with blue markers concentrated near the vertices. These patterns strongly support ILS as the dominant driver of topological discordance, while the residual discordance (QC/QD/QI = 1/0/1) likely reflects secondary processes like hybridization.

**Figure 5:**
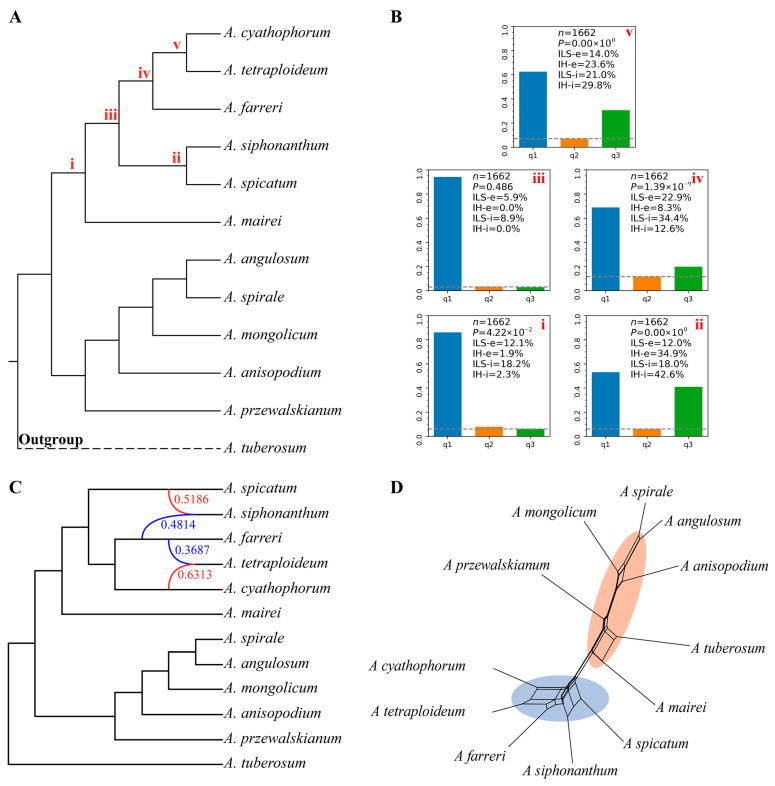
The detection of the ILS and reticulate evolution signals and network reconstruction. (**A**) The species phylogeny reconstructed from 1662 SCGs in subg. *Cyathophora*. (**B**) The signals of incomplete lineage sorting (ILS-i) and introgression/hybridization (IH-i) across the key phylogenetic nodes (red i–v) are identified and visualized, corresponding to the nodes in the phylogenetic tree. (**C**) The maximum pseudolikelihood species network, inferred using PhyloNet. The red and blue lines indicate greater and lesser parental contributions, respectively, to the hybrid lineages. The numbers adjacent to the branches indicate inheritance probabilities (*r*) for each hybrid node. (**D**) the reticulate phylogeny reconstructed through NANUQ topological analysis. The colored ovals highlight areas of possible reticulation.

Phytop analyses revealed pronounced incomplete lineage sorting signatures (ILS-i; 8.9–34.4%) across subg. *Cyathophora* lineages, with peak ILS-i values (34.4%) at the *A. farreri* and *A. tetraploideum*–*A. cyathophorum* divergence nodes (Figure 5A,B, node (iv)). Conversely, four nodes ((i), (ii), (iv), (v)) exhibited substantial introgression probability (IH-i: 2.3–42.6%), with the highest IH-i probability at the *A. spicatum*–*A. siphonanthum* divergence node (42.6%; Figure 5A,B, node (ii)).

### 2.6. Reticulation Identification

To evaluate reticulate evolution contributions to phylogenetic discordance in subg. *Cyathophora*, we reconstructed phylogenetic networks using the NANUQ methodology. SNaQ analyses showed decreasing log-pseudolikelihood values with increasing hybridization parameters (*h* = 0–5), and the optimal network was found at *h* = 2 (Table S3). This optimal network indicates two hybridization events within *Cyathophora* lineages (Figure 5C): (i) *A. tetraploideum* originates from *A. farreri* (36.87%) and *A. cyathophorum* (63.13%); (ii) *A. siphonanthum* exhibits near-equal genomic contributions to *A. spicatum* (51.86%) and *A. farreri* (48.14%). The NANUQ network reconstruction confirmed discernible reticulation nodes connecting four core taxa (Figure 5D), demonstrating the critical role of hybridization in shaping the subgenus’ evolutionary history.

## 3. Discussion

### 3.1. Phylogenomic Data Revealed New Species Relationships in Subg. Cyathophora

Phylogenomic data are widely used for phylogenetic analyses and have proven highly efficient for resolving evolutionary relationships in genus *Allium* [11,13,14,16,29,30]. Our results confirm the monophyly of subg. *Cyathophora* [10,11,16,31], and identify *A. siphonanthum* as a new member with novel species relationships. However, significant phylogenetic discordance emerged between the single-copy genes (SCGs) and plastid data. Multiple studies have indicated that factors such as incomplete lineage sorting (ILS), gene introgression, or horizontal gene transfer can sometimes obscure rather than clarify species relationships [27,32,33,34]. For instance, Ma et al., 2024 [33], analyzed *Quercus* species relationships using both plastid and transcriptome data. The plastid-based tree showed highly confused interspecific relationships, whereas transcriptome data clearly distinguished all species. Moreover, our coalescent-based analyses of transcriptomic analyses yielded significantly stronger nodal support than the plastid phylogenies. Therefore, we propose that nuclear-derived phylogenies are more reliable than those derived from plastid data.

Building upon prior molecular studies [10,11,16], our analyses confirm *A. mairei* as the earliest diverging lineage in this subgenus (UFBS/BS = 100; LPP/BS = 1.00/100). This placement is further corroborated by its diagnostic inflorescence: unequal pedicels bearing few flowers with patent trumpet-shaped corollas, contrasting with the uniform pedicels and densely flowered umbels of other *Cyathophora* species (Figure 1, Table 1) [10]. Furthermore, we discovered that *A. siphonanthum*, which has long been neglected and whose systematic position remained unknown, was shown to be a member of the subg. *Cyathophora* and exhibits a sister species relationship with *A. spicatum*. Morphologically, these two species show a high degree of morphological congruence, notably characterized by their strongly abbreviated pedicels, white to purple-red perianth segments, and both their stamens and styles exceeding the perianth (Figure 1, Table 1). Field investigations further indicate their shared ecological affinity for arid hillslopes, where they develop extensive root systems to withstand seasonal drought stress. It has also been suggested that the conspicuously elongated spicate inflorescence of *A. spicatum* compared to the umbel in its congeners represents an alternative strategy to adapt to the extreme alpine habitat, accompanied by consequential changes in seed size, flowering phase, and ovary size [10,17]. *Allium cyathophorum* forms a sister relationship with *A. tetraploideum*, and these two species cluster with *A. farreri*. All three taxa exhibit purple to dark maroon perianth segments, though conspicuous differences exist in basal perianth morphology, inner filament characteristics, and micro-morphological features (including anticlinal wall patterns, pollen morphology, and stomatal apparatus) [17]. The most pronounced distinction lies in their karyotypes: both *A. cyathophorum* and *A. farreri* are diploid (2n = 16), whereas *A. tetraploideum* is tetraploid (2n = 32). Li et al., 2021 [11], demonstrated that *A. tetraploideum* originated from hybridization between the two diploids *A. farreri* and *A. cyathophorum* or their close relatives, potentially involving an extinct diploid progenitor. Thus, our integrated morphological and molecular analyses establish *A. siphonanthum* as a new member of the subg. *Cyathophora* and resolve novel phylogenetic relationships within this subgenus.

### 3.2. Underlying Causes for Phylogenetic Discordances in Subg. Cyathophora

Substantial phylogenomic evidence indicates that phylogenetic discordance primarily stems from incomplete lineage sorting (ILS) and reticulate processes like introgression or hybridization [27,35,36,37]. In subg. *Cyathophora*, we detected pronounced incongruence not only among individual gene trees but also between SCGs- and plastid-derived species trees (Figure 1, Figure 2 and Figure 3). Integrative analyses demonstrate that ILS is a primary driver of discordance, supported by two lines of evidence: First, there are significant positive correlations (*p* < 0.001) between branch lengths and concordance factors (gCF and sCF) (Figure 4), consistent with established ILS-associated branch length variations and phylogenetic discordance [27,36,38,39]. Second, systematic quantification reveals substantial ILS signals (ILS-i ranging from 8.9% to 34.4%, Figure 5), with qcCFs statistics confirming ILS as the predominant source of topological conflicts (Figure 3 and Figure S5). Notably, maximal bootstrap support (BS = 100) at short branch nodes (Figure 4D,H) represents a characteristic signature of ILS-mediated discordance documented across diverse taxa [27,28,38,40]. Meanwhile, ILS has been recognized as a major factor influencing species relationships and evolution [27,33,34]. Here, ILS not only contributes significantly to the phylogenetic discordance within this subgenus but may also drive species adaptation to diverse environments via the maintenance of adaptive loci. For instance, *A. spicatum* has evolved a unique spicate inflorescence, enhancing its adaptation to the arid western QTP, while *A. cyathophorum*, *A. farreri*, and *A. tetraploideum* have developed purple flowers that attract more insect pollinators, increasing fruit set rates. However, further investigation using expanded samples across broader geographical ranges is needed to understand how the maintenance of adaptive loci enhances environment adaption.

Complementary to ILS-mediated discordance, our analyses revealed definitive reticulate evolution signals in subg. *Cyathophora* contributing to phylogenetic incongruences, a phenomenon widely documented across eukaryotes [35,41,42]. Alongside marked phylogenetic discordance within subg. *Cyathophora*, we identified quantifiable hybridization/introgression signals (IH-i values spanning 2.3–42.6% across four specific nodes; Figure 5B). These findings demonstrate a causal relationship between reticulate evolutionary processes and the observed phylogenetic incongruence. Quartet Sampling analysis further revealed robust evidence of ancient hybridization in subg. *Cyathophora*, with key nodes showing maximal nodal support (UFBS/BS = 100, LPP = 1.0) and high QC values (>0.8) (Figure 1, Figure 2 and Figure 3), yet paradoxically low quartet differential values (QD <0.25/NA), a pattern consistent with deep reticulation events [43].

Furthermore, SNaQ analysis identified two hybridization events (Figure 5C): (i) *A. tetraploideum* originated from hybridization between *A. cyathophorum* and *A. farreri*; (ii) the newly described *A. siphonanthum* derived from hybridization between *A. spicatum* and *A. farreri*. These findings align with Li et al., 2019 [17], who proposed *A. tetraploideum*’s hybrid origin involving *A. farreri*, *A. cyathophorum*, and potentially an extinct diploid progenitor. Morphological and cytological evidence [10,11,17] also provide robust support for interspecific hybridization. Furthermore, we identified novel hybridization events within subg. *Cyathophora*, including the new member *A. siphonanthum* originating from hybridization between *A. spicatum* and *A. farreri*. This hybrid origin was also supported by the discordant phylogenetic results, where *A. siphonanthum* is sister to *A. spicatum* in the SCGs-based tree, whereas it clusters with the lineage of *A. spicatum* + *A. farreri* in the plastid tree (Figure 3). These hybridization events were also corroborated by the NANUQ network topology. Numerous studies have suggested that recurrent hybridization events drive reticulate evolution [44,45]. Although such processes can confound phylogenetic interpretations, as evidenced by the significant phylogenetic discordance and reticulate evolution, they simultaneously confer evolutionary advantages through adaptive introgression, enhancing both species diversity and environmental adaptability [46,47]. The species of subg. *Cyathophora* predominantly inhabit the QTP and HDMs, biodiversity hotspots shaped by intricate topography and dynamic climatic conditions [48,49,50,51,52]. Previous studies have documented pervasive adaptive introgression during the speciation processes of taxa within this region [42,44,53,54,55]. In *Allium*, many polyploids have been reported in this region, such as *A. wallichii*, *A. przewalskianum*, and *A. sikkimense*, which are correlated with the area’s geographical orogenies and climatic fluctuations [10,11,13,29,56,57]. In addition, *Allium* species possess adaptive traits, including a herbaceous habit (enabling rapid generations), perennial growth (extending lifespan), clonal reproduction (via bulb/rhizome), and stress tolerance, which may facilitate hybridization and environmental adaptation [58,59]. Our phylogeographic analyses indicate that initial subgenus divergence coincided with the HDMs’ orogenic uplift (4–3 million years ago, Ma), with subsequent Pleistocene climatic fluctuations amplifying species divergence and niche differentiation [10]. This evidence collectively indicates that hybridization in subg. *Cyathophora* is evolutionarily linked to the regional orogeny and paleoclimate, representing a key adaptive strategy that facilitates species divergence and environmental adaptation within the harsh ecosystems of the QTP and HDMs. In summary, the significant phylogenetic discordance in subg. *Cyathophora* results from incomplete lineage sorting (ILS) and reticulate evolution, potentially driven by past orogenic events and climatic fluctuations within the QTP and HDMs.

## 4. Materials and Methods

### 4.1. Sampling, Morphological Observation, and Transcriptome Sequencing

Over the last decade, we carried out extensive field surveys across the QTP and HDMs and collected numerous samples of subg. *Cyathophora* and relatives. The morphological characteristics of all species within this subgenus were documented through literature surveys and field observations. Fresh leaves of each species in full bloom were gathered, and the detailed collection protocol comprised the following steps: preparing sterilized tools (tweezers, foil, marker); selecting healthy, pest/disease-free leaves; briefly rinsing samples with sterile water followed by drying with absorbent paper; securely wrapping samples in foil with white cloth tape; labeling with marker; and immediately transferring specimens to liquid nitrogen. The samples were then transported into an −80 °C freezer until they underwent RNA isolation and sequencing processing. The morphologies of the species in subg. *Cyathophora* were also photographed in the field. We also downloaded the transcriptome data of subg. *Rhizirideum* species from public databases (National Center for Biotechnology Information/NCBI and National Genomics Data Center/NGDC), including *Allium angulosum* L., *Allium spirale* Willd., *Allium mongolicum* Regel, *Allium anisopodium* Ledeb., *Allium przewalskianum* Regel, and *Allium tuberosum* Rottler ex Spreng. A total of 12 species were collected, and the detailed sample information is listed in Table S4. All collected specimens were identified by *Allium* taxonomy experts Xing-Jin He, Min-Jie Li, and Deng-Feng Xie to ensure accurate species identification.

Transcriptomic sequencing began with RNA isolation using the RNAprep Pure Plant Kit (Tiangen Biotech, Beijing, China). RNA quality was verified using NanoPhotometer spectrophotometry (IMPLEN, Munich, Germany) and an Agilent 2100 Bioanalyzer (Santa Clara, CA, USA) assessment. Sequencing libraries were then constructed with the NEB Next Ultra™ RNA Library Prep Kit (Illumina) (Ipswich, MA, USA), and sequenced for 150 bp paired-end reads on an Illumina NovaSeq 6000 platform. Raw reads were processed using fastP [60] and Trimmomatic v0.36 [61] to remove adapters and low-quality sequences, generating cleaned reads for transcriptome assembly. All raw data were deposited in the Genome Sequence Archive (GSA: NGDC) under accession codes detailed in Table S4.

### 4.2. Transcriptome Assembly and Low-Copy Gene Identification

Paired-end clean reads from each transcriptome were assembled de novo using Trinity v.2.1.5 [62] with default parameters. TransDecoder v.5.0.2 (http://transdecoder.sourceforge.net/) (accessed on 16 March 2025) was utilized to predict the longest putative CDS and protein sequences within open reading frames (ORFs). CD-HIT v4.6.1 [63] was employed to generate non-redundant CDS and protein sequences, applying a threshold of 0.95 for sequence identity. To identify single-copy genes (SCGs), we conducted orthogroup inference with OrthoFinder v2.5.2 [64] to identify single-copy genes (SCGs). Each SCG was aligned with PRANK [65], using the ‘-codon’ parameter to guide the alignment. Finally, sequences shorter than 300 bp or containing more than 50% gaps were removed, resulting in a final set of 1662 SCGs for the subsequent analyses.

### 4.3. Phylogenetic Reconstruction and ICA Score Calculation

Phylogenetic reconstruction was conducted using concatenation- and coalescent-based approaches on the curated dataset of 1662 single-copy core genes (SCGs). Initial sequence processing involved alignment with MAFFT v7.505 [66] using default parameters, followed by the refinement of ambiguous regions through TrimAl v1.2 [67] with a 0.8 gap threshold (−gt). For concatenation analysis, a supermatrix was constructed and analyzed under the maximum likelihood (ML) framework in IQ-TREE v1.6.8 [68]. ModelFinder [69] determined the optimal codon-specific partitioning schemes and substitution models; nodal support was evaluated with 1000 ultra-fast bootstrap (UFBS) replicates (−bb 1000). Concurrently, coalescent-based species tree estimation was employed using ASTRAL-III v5.6.3 [70] and two complementary strategies: (i) the comprehensive integration of gene trees with local posterior probabilities (LPPs) for nodal confidence under ‘−t 3’ configuration [71]; (ii) species tree reconstruction using optimized ML gene trees with multilocus branch support derived from 1000 bootstrap replicates (−i −b −r parameters). All gene trees were independently generated in IQ-TREE using ModelFinder-optimized substitution models, with each SCG’s phylogenetic uncertainty quantified through 1000 rapid bootstrap replicates of unpartitioned alignments.

Phylogenetic concordance analysis was conducted using phyparts v0.01 [72,73] through systematically comparing gene tree and species tree topologies. The Internode Certainty All (ICA) values were calculated to evaluate nodal support or conflict for each lineage. These ICA values were interpreted as follows: values approaching 1 indicated strong internodal consensus; values near 0 suggested equal support for one or more conflicting bipartitions; and negative values signified dominant conflicting bipartition frequencies [72].

### 4.4. Whole-Genome Resequencing, Plastome Assembly, and Phylogenetic Analyses

To perform phylogenetic analyses based on plastome data, genomic DNA was extracted from fresh or silica-gel-dried materials using the CTAB method [74], followed by whole-genome resequencing on the Illumina HiSeq 6000 platform (Novogene, Beijing, China). Post-sequencing quality assessment yielded clean data for plastome assembly through GetOrganelle v1.7.7.1 [75], with subsequent annotation implemented in GeSeq (https://chlorobox.mpimp-golm.mpg.de/geseq.html) (accessed on 12 March 2025). The annotated plastid genomes underwent rigorous manual verification and refinement using Geneious v11.0.5 (https://www.geneious.com/), ensuring annotation accuracy across all samples. All 12 plastomes used in this study are listed in Table S2.

We further extracted the 107 genes and 43 intergenic spacers (IGSs) from the plastomes for phylogenetic analyses. Sequence processing comprised alignment with MAFFT v7.505 [66] under default parameters, supplemented by precision trimming of ambiguous regions through TrimAl v1.2 [67] with optimized parameters (−gt 0.8). Two complementary phylogenetic approaches were employed: (i) concatenation-based maximum likelihood analysis executed in IQ-TREE v1.6.8 [68] with 1000 ultra-fast bootstrap (UFBS) replicates; (ii) coalescent-based species tree estimation conducted in ASTRAL-III v5.6.3 [70] incorporating bootstrap (BS) support and local posterior probabilities (LPPs). Nodal robustness assessment included Internode Certainty All (ICA) quantification [72,73] applied to key phylogenetic nodes. Methodological consistency with the transcriptomic analysis protocols (Section 4.3) was maintained, and cross-dataset parameter standardization ensured comparative analytical validity.

### 4.5. Phylogenetic Discordance Analysis

We further evaluated the phylogenetic discordance among gene trees as well as between nuclear and plastid species trees using two methods: (i) Concordance factor analysis using IQ-TREE v2.1.3 [68] to calculate gene (gCF) and site (sCF) concordance factors, quantifying, respectively, the proportion of supporting gene trees (gCF) and aligned sites (sCF) per branch (methodology details: http://iqtree.org) (accessed on 21 March 2025) [76]. An R script (https://robertlanfear.com/blog/files/concordance_factors.html) (accessed on 21 March 2025) visualized gCF-sCF correlations with bootstrap support and branch lengths. (ii) The Quartet Sampling method [77] was implemented via the quartet_sampling.py script (https://github.com/fephyfofum/quartetsampling) (accessed on 21 March 2025) with 1000 bootstrap replicates. This approach generated three core nodal metrics: (i) quartet concordance (QC), quantifying topological congruence between the sampled quartets and candidate species tree; (ii) quartet differential (QD), measuring phylogenetic conflict asymmetry, where skewed distributions may indicate introgression events; (iii) quartet informativeness (QI), evaluating phylogenetic signal strength through likelihood-based comparisons of alternative topologies. To assess terminal node reliability, quartet fidelity (QF) values were calculated to detect unstable “rogue” taxa.

### 4.6. Estimation of Incomplete Lineage Sorting

To assess whether phylogenetic discordance between nuclear–plastid phylogenies and among individual gene trees could be attributed to incomplete lineage sorting (ILS), we implemented a quartet-based gene tree summarization framework. This methodology calculated quartet count concordance factors (qcCFs) and derived corresponding *p*-values to statistically evaluate the congruence between the gene trees and species tree topologies [78]. Specifically, the quartetTreeTestInd function from the R package MSCquartets v1.0 [79] was applied to analyze all 1662 SCG trees. The resultant qcCFs were visualized through tetrahedral simplex plots within two distinct phylogenetic models: T1 (incorporating the species tree reconstructed by ASTRAL-III) and T3 (without species tree assumptions). In these visualizations, color-coded symbols (red = rejection, blue = non-rejection at α thresholds: 0.01, 0.001, 1 × 10^−4^, 1 × 10^−6^) represented individual quartet vectors, where centroid-proximal distributions supported ILS dominance, while vertex-adjacent clustering suggested alternative evolutionary processes such as introgression.

To comprehensively characterize incomplete lineage sorting (ILS) and introgression/hybridization (IH) signals across lineages, we implemented the phylogenomic analysis pipeline in Phytop v1.0 [80], which quantitatively assesses ILS/IH magnitudes and visualizes spatial patterns based on ASTRAL-generated gene trees. A curated dataset was prepared by extracting SCGs for 12 representative taxa from the original 1662 SCG collection. Each gene tree was reconstructed using IQ-TREE v1.6.8 under optimal substitution models as detailed in Section 4.3. The species tree topology was subsequently inferred through ASTRAL-III v5.6.3 with the branch annotation parameter (−t 2). This consensus species tree served as the scaffold for Phytop’s comparative analysis, enabling the systematic quantification and heatmap visualization of lineage-specific ILS/IH values through its integrated topological permutation algorithms.

### 4.7. Reticulate Evolution Detection

To systematically evaluate the contribution of reticulate evolution (hybridization/introgression) to observed phylogenetic discordance, we implemented a dual analytical framework combining phylogenetic network reconstruction and quartet-based distance methods. First, maximum pseudolikelihood network inference was performed using PhyloNet v3.8.0 [81] through its SNaQ algorithm [82]. This analysis utilized 1662 SCG trees to test evolutionary models permitting 0–5 reticulation events (*h*). We conducted 100 independent optimizations per *h*-value to identify maximum likelihood network configurations, and final topologies were visualized in Dendroscope v3.8.2 [83]. To address uncertainties in pseudolikelihood model selection, we complemented this approach with the NeighborNet Using Quartet distance (NANUQ) method [84]. Empirical quartet counts from SCG trees were processed through MSCquartets’ NANUQ function under the T3 model (α = 0.01) to calculate taxon-pair network distances. These distances were subsequently transformed into a consensus split graph using SplitsTree4 v4.15.1′s NeighborNet algorithm [85], enabling the simultaneous visualization of conflicting phylogenetic signals through the multidimensional scaling of quartet discordance patterns.

## 5. Conclusions

In this study, we generated transcriptomic and plastid datasets for subg. *Cyathophora* species and relatives. From these, 1662 single-copy nuclear genes (SCGs) and 150 plastid loci were extracted for phylogenetic reconstruction using both concatenation- and coalescent-based methods, with their integrated morphological characteristics used to assess interspecific relationships. Our analyses definitively resolve *Allium siphonanthum*, a taxon with historically uncertain placement, within subg. *Cyathophora*. While novel species relationships were resolved, significant phylogenetic discordance was detected. Multiple pieces of evidence indicate that this discordance stems from incomplete lineage sorting (ILS) and/or hybridization, processes likely facilitated by intense orogeny and paleoclimatic fluctuations across the HDMs and QTP. These findings substantially advance our understanding of diversification mechanisms in this subgenus.

## Figures and Tables

**Table 1:** Main morphological characteristics of species in subg. *Cyathophora*.

Species	*Allium cyathophorum*	*Allium tetraploideum*	*Allium farreri*	*Allium siphonanthum*	*Allium spicatum*	*Allium mairei*
bulb	solitary or clustered, cylindric	solitary or clustered, cylindric	solitary or clustered, cylindric	solitary or clustered, cylindric	solitary, cylindric	usually clustered, cylindric
bulb tunics	grayish brown, fibrous	grayish brown, fibrous	grayish brown, fibrous	yellowish brown, subreticulate	yellowish brown, fibrous	yellowish brown to grayish brown, fibrous
scape shape	terete, usually 2-angled, covered with leaf sheaths only at base	terete, usually 3-angled, covered with leaf sheaths only at base	terete, usually 2-angled, covered with leaf sheaths only at base	terete, not angled, covered with leaf sheaths only at base	terete, hollow, not angled, covered with leaf sheaths only at base	terete, 2-angled, covered with leaf sheaths only at base.
scape length	13–25 cm	13–25 cm	13–25 cm	18–60 cm	5–40 cm	10–30 cm
leaf	shorter than scape	shorter than scape	shorter than scape	subequal to scape	subequal to scape	shorter than or subequal to scape
inflorescence	umbel hemispheric, laxly flowered	umbel hemispheric, laxly flowered	umbel hemispheric, laxly flowered	umbel globose, densely many flowered	spike, densely many flowered	umbel, with very few flowers
pedicel length	equal, 1–3 × as long as perianth, ebracteolate	equal, 1–3 × as long as perianth, ebracteolate	equal, 1–3 × as long as perianth, ebracteolate	equal, shorter than 1/3 of perianth, ebracteolate	equal, shorter than 1/3 of perianth, ebracteolate	unequal, 1.5–2 × as long as perianth, ebracteolate
spathe	1(–3)-valved, persistent	1(–3)-valved, persistent	1(–3)-valved, persistent	2-valved, persistent	1-valved, persistent	1-valved, persistent
perianth	purple, retuse to obtuse at apex	dark maroon, retuse to obtuse at apex	purple, acuminate at apex	white to purple-red, retuse to obtuse at apex	white to purple-red, retuse to obtuse at apex	pale red to purple-red, apex obtuse or acute
inner filaments	shoulder-shaped at base, no teeth	shoulder-shaped at base, no teeth	triangular at base, no teeth	wide at base, entire or 1-toothed on each side	wide at base, entire or 2-toothed on each side	conical at base, no teeth
style	shorter than ovary	shorter than ovary	shorter than ovary	longer than ovary	longer than ovary	shorter than ovary
chromosome number	2n = 16	2n = 32	2n = 16	unknown	2n = 16	2n = 16, 32
flowering season	June to August	June to August	June to August	September to October	August to October	August to October

## Data Availability

All sequencing data have been deposited in the Genome Sequence Archive (GSA) of the National Genomics Data Center (NGDC) under project PRJCA039276.

## Supplementary Materials

The following supporting information can be downloaded at: https://www.mdpi.com/article/10.3390/plants14132083/s1, Figure S1: The concordance and conflict statistics for the 1662 SCG trees. The numbers above and below the branches indicate the counts of gene trees concordant with and in conflict with the species tree, respectively. The pie charts at the main clades show the proportion of genes in concordance (blue) and conflict (green = a single dominant alternative; red = all other conflicting trees); Figure S2: The concordance and conflict statistics for the 150 plastid loci trees. The numbers above and below the branches indicate the counts of gene trees concordant with and in conflict with the species tree, respectively. The pie charts at the main clades show the proportion of genes in concordance (blue) and conflict (green = a single dominant alternative; red = all other conflicting trees); Figure S3: The bootstrap value (BS), gene concordance factor (gCF), and site concordance factor (sCF) for each node across the species tree based on the 1662 SCGs trees. The numbers above the branches represent the values of BS/gCF/sCF; Figure S4: The bootstrap value (BS), gene concordance factor (gCF), and site concordance factor (sCF) for each node across the species tree based on the 150 plastid loci trees. The numbers above the branches represent the values of BS/gCF/sCF; Figure S5: Simplex plots of qcCFs under the MSC model of ILS with the T1 and T3 models based on the 1662 LCGs trees. These analyses were performed under different rejection levels (from 0.01 to 1 × 10^−6^). The red triangles in the plot indicate the rejection of the MSC on the species tree, while the blue circles indicate probable ILS; Table S1: The ten transcriptomes’ assembly statistics; Table S2: The 12 chloroplast genomes’ information collected in this study; Table S3: The network scores (−ploglik) estimated in the SNaQ analysis. The optimal network in each analysis is indicated in bold; Table S4: All transcriptomes, locations, and voucher information used in this study.
