# Supplementary material for: Phylogenomic Analyses Reveal Species Relationships and Phylogenetic Incongruence with New Member Detected in Allium Subgenus Cyathophora
Source: Plants (Basel). 2025 Jul 7;14(13):2083. doi: 10.3390/plants14132083 (PMC12252102; doi:10.3390/plants14132083)
Supplement: Supplementary file 1 [file plants-14-02083-s001.zip › plants-3634690-supplementary.pdf]

Supplementary Materials

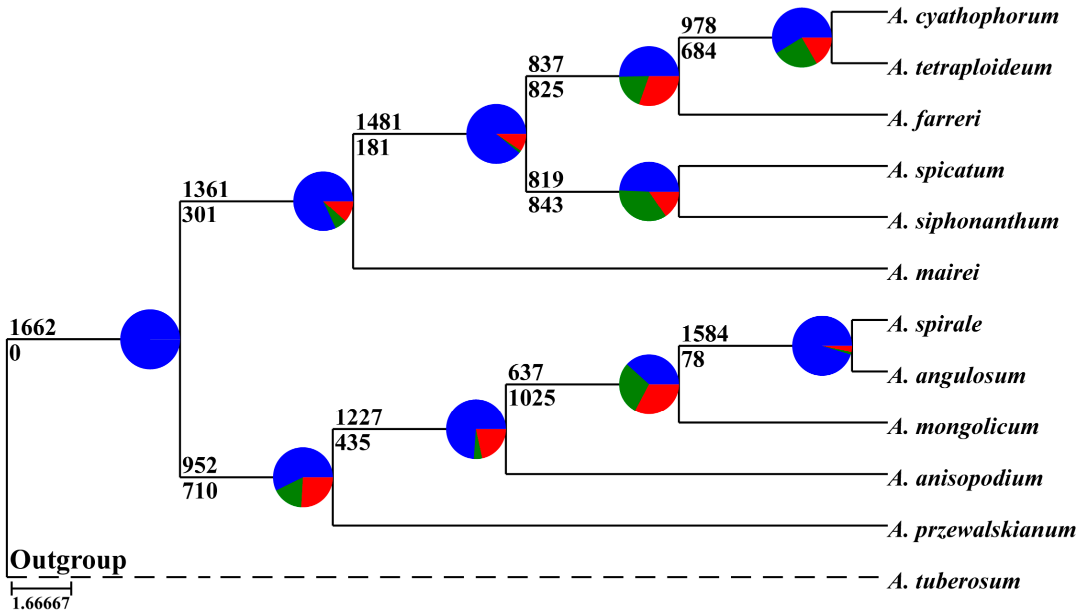

Figure S1. Concordance and conflict statistics for 1,662 SCG trees. Numbers above and below the branches indicate the count of gene trees concordant with and in conflict with the species tree, respectively. The pie charts at main clades show the proportion of genes in concordance (blue) and conflict (green = a single dominant alternative; red = all other conflicting trees).

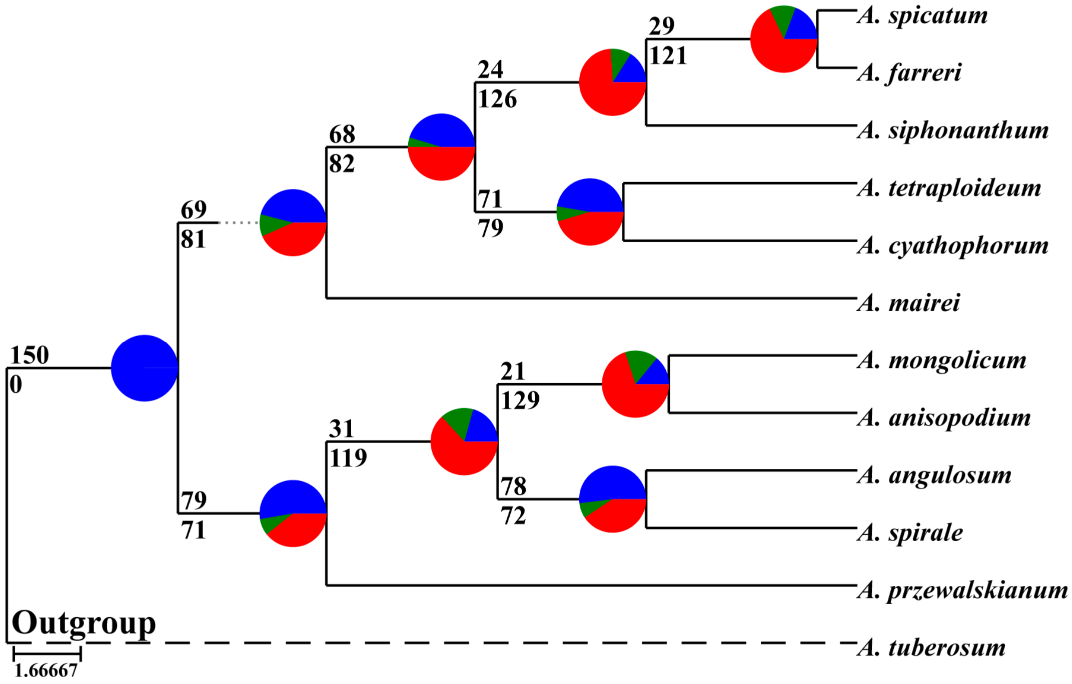

Figure S2. Concordance and conflict statistics for 150 plastid loci trees. Numbers above and below the branches indicate the count of gene trees concordant with and in conflict with the species tree, respectively.The pie charts at main clades show the proportion of genes in concordance (blue) and conflict (green = a single dominant alternative; red = all other conflicting trees).

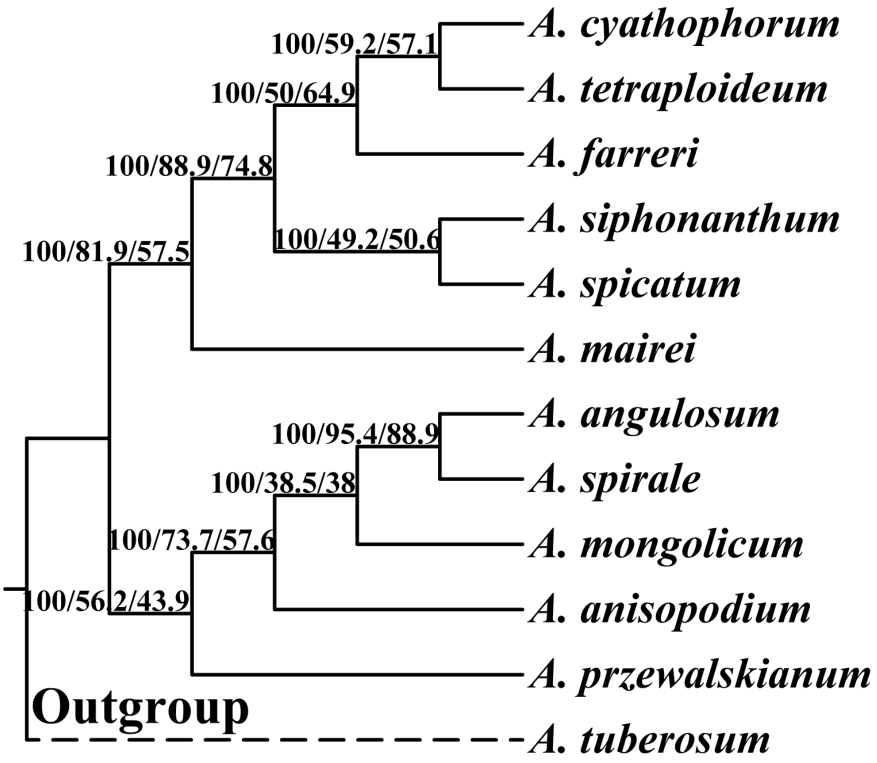

Figure S3. Bootstrap values (BS), gene concordance factor (gCF) and the site concordance factor (sCF) for each node across the species tree based on 1,662 SCGs trees. The numbers above the branches represent the values of BS/gCF/sCF.

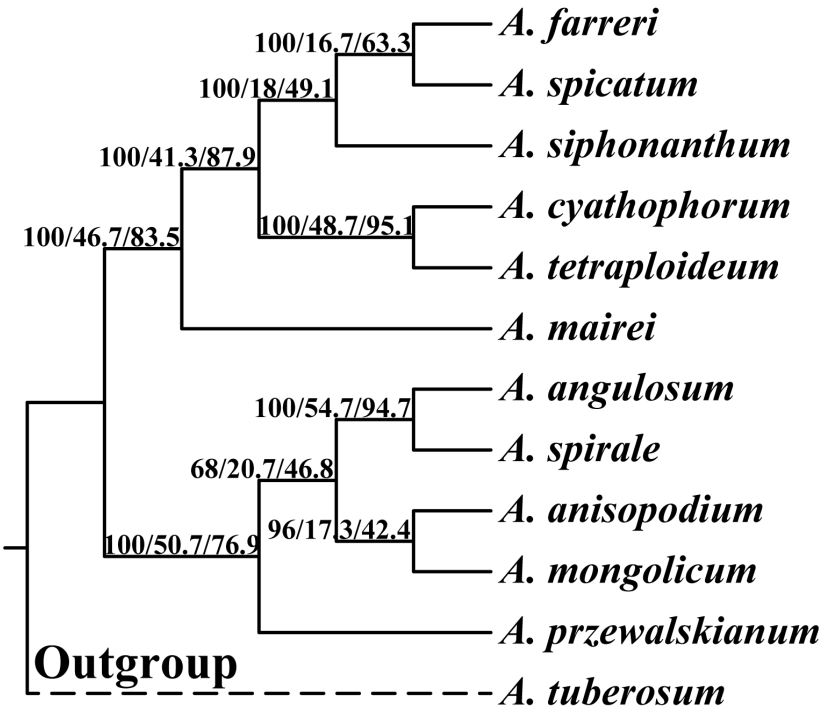

Figure S4. Bootstrap values (BS), gene concordance factor (gCF) and the site concordance factor (sCF) for each node across the species tree based on 150 plastid loci trees. The numbers above the branches represent the values of BS/gCF/sCF.

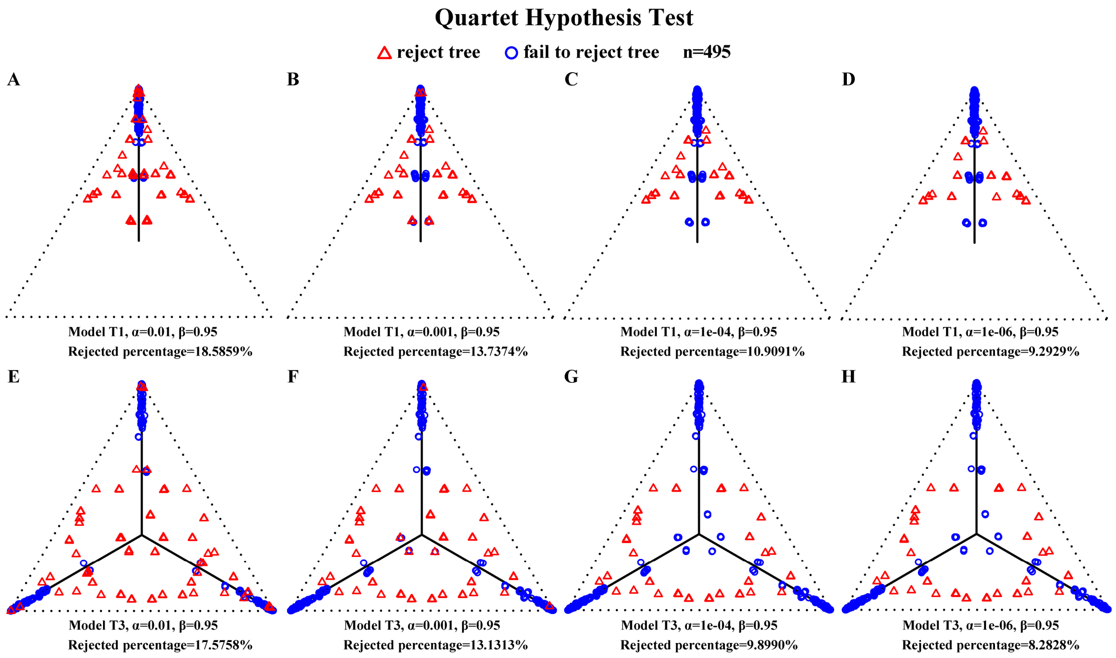

Figure S5. Simplex plots of qcCFs under the MSC model of ILS with T1 and T3 models based on the 1,662 LCGs trees. The analyses were performed under different rejection levels (from 0.01 to 1e-06). Red triangles in the plot indicate rejection of the MSC on the species tree, while blue circles indicate probable ILS.

Table S1. The 12 transcriptomes assembly statistics.

Species	Assembled bases	Mean transcript length	Transcripts	N50	GC percentage
A. cyathophorum	23287299	834	30760	1131	41.8
A. tetraploideum	23378919	819	30800	1098	42.15
A. farreri	22409742	872	29019	1188	41.77
A. siphonanthum	24353247	782	35307	1041	41.88
A. spicatum	22791528	871	28042	1191	41.91
A. mairei	28906896	791	41756	997	42.81
A. angulosum	22858446	863	28967	1107	41.62
A. spirale	19713114	863	24201	1125	41.67
A. mongolicum	26149632	892	32449	1245	41.83
A. anisopodium	23749320	814	31588	1014	41.69
A. przewalskianum	23756484	847	31810	1149	41. 94
A. tuberosum	24751824	814	34186	1104	41. 39

Table S2. The 12 chloroplast genomes information collected in this study.

**Species**	**Size(bp)**	**GC(%)**	**Gene region(bp)**	**CDS length(bp)**	**tRNA length(bp)**	**Gene number**	**NCBI numbers**
A. angulosum	153590	36.85	90534	78648	2834	131	OR605563
A. anisopodium	153407	36.84	91078	79167	2859	131	NC_068830
A. cyathophorum	152975	36.86	90934	79020	2864	132	MN882559
A. farreri	153484	36.85	91207	79293	2864	132	MN882560
A. mairei	153956	36.85	91002	79089	2863	132	MN882561
A. mongolicum	153667	36.78	91066	79155	2859	131	ON008504
A. przewalskianum	153243	36.87	91152	79239	2863	132	MN882562
A. siphonanthum	153724	36.86	89066	77221	2795	131	WX296797
A. spicatum	152876	36.88	91191	79278	2863	132	MN882563
A. spirale	153549	36.83	91036	79125	2859	131	ON008502
A. tetraploideum	152906	36.86	90898	78984	2864	132	MN882564
A. tuberosum	153550	36.93	90539	78657	2830	131	OR605584

Table S3. Network scores (-ploglik) estimated in the SNaQ analysis. The optimal network in each analysis is indicated in bold.

	hmax	-Ploglik score		hmax	-Ploglik score
A	0	-117504.6584	B	0	-117583.9279
	1	-116509.8417		1	-116580.3809
	**2**	**-116316.9033**		**2**	**-116398.1459**
	3	-116294.3994		3	-116340.2424
	4	-116340.3209		4	-116509.6433
	5	-116299.2231		5	-116490.234
	hmax	-Ploglik score		hmax	-Ploglik score
C	0	-117584.7098	D	0	-117763.4988
	1	-116589.815		1	-116660.7633
	**2**	**-116476.5536**		**2**	**-116509.7055**
	3	-116340.274		3	-116360.5973
	4	-116509.7043		4	-116583.8028
	5	-116492.9189		5	-116509.8188
	hmax	-Ploglik score			
E	0	-117916.3828			
	1	-116675.144			
	**2**	**-116589.0688**			
	3	-116509.7868			
	4	-116589.0572			
	5	-116588.8279			

Table S4. All transcriptomes, location and voucher information used in this study.

Species	NCBI or CNCB numbers	Location	Latitude (°)	Longitude (°)	Altitude (m)	Voucher number (herbarium)	Voucher collector	Collect date	Habitat
A. cyathophorum	SRR10828153	Mangkang, Tibet	N29.7248	E98.5316	3805	LMJ2013082880 (SZ)	Li MJ, Xie DF	2013-8-28	alpine meadow
A. tetraploideum	SRR10828151	Tongde, hebei, Qinghai	N34.7619	E100.8930	3329	LMJ2013071716 (SZ)	Li MJ, Xie DF	2013-7-17	alpine meadow
A. farreri	SRR10828152	Luqu, langmuqiao,GanSu	N34.9405	E102.5669	2800	lmj2013081472 (SZ)	Li MJ, Xie DF	2013-8-14	alpine meadow
A. siphonanthum	PRJCA039276	Zhongdian, Yunnan	N27.1637	E100.0912	1914	XDF2023071809 (SZ)	Xie DF, Cai J	2023-7-18	scrub of a dry-hot valley
A. spicatum	SRR10828150	Lhasa, KitayMa, Tibet	N29.9774	E91.6798	3596	LMJ2013090390 (SZ)	Li MJ, Xie DF	2013-9-3	alpine meadow
A. mairei	PRJCA039276	Ninglang, YunNan	N27.2812	E100.9216	2538	XDF2023090814 (SZ)	Xie DF, Wang Y	2023-9-8	forest edge
A.przewalskianum	SRR10828148	Tongde, hebei, Qinghai	N34.7619	E100.8930	3329	lmj2014092423 (SZ)	Li MJ, Xie DF	2013-7-17	alpine meadow
A.angulosum	SRR9077156	—	—	—	—	—	—	—	—
A. spirale	CRR301241	—	—	—	—	—	—	—	—
A. mongolicum	SRR8704495	—	—	—	—	—	—	—	—
A. anisopodium	SRR29002603	—	—	—	—	—	—	—	—
A. tuberosum	SRR25211041	—	—	—	—	—	—	—	—

SZ: herbarium of sichuan university; the '—' represent the missing sample information because the transcriptome data come from public databases.
